# Comparison of first-line chemotherapy regimens for ovarian carcinosarcoma: a single institution case series and review of the literature

**DOI:** 10.1186/s12885-018-4082-6

**Published:** 2018-02-09

**Authors:** Melissa Brackmann, Marina Stasenko, Shitanshu Uppal, Jake Erba, R. Kevin Reynolds, Karen McLean

**Affiliations:** 1 Division of Gynecologic Oncology, Department of Obstetrics and Gynecology, University of Michigan, Ann Arbor, USA; 2 University of Michigan, 1500 E. Medical Center Drive, Ann Arbor, MI 48109-5276 USA

**Keywords:** Ovarian carcinosarcoma, MMMT, Chemotherapy, Outcomes, Carboplatin, Ifosfamide

## Abstract

**Background:**

The optimal first-line chemotherapy for ovarian carcinosarcoma has not yet been determined. We therefore sought to determine the progression-free survival (PFS) and overall survival (OS) for patients with ovarian carcinosarcoma treated at our institution with different first-line chemotherapy regimens.

**Methods:**

This single-institution, retrospective analysis included all patients with ovarian or primary peritoneal carcinosarcoma diagnosed from September 1996 to July 2017. Kaplan Meier analysis with a log-rank Mantel-Cox test was used to compare PFS and OS between treatment groups, and a *p*-value of < 0.05 was considered statistically significant.

**Results:**

Thirty-one patients met inclusion criteria: two patients were stage IC, 5 were stage II, 21 were stage III, and 3 were stage IV. The median PFS and OS for all stages was 9.3 and 19.7 months respectively. Fifteen patients (48%) received carboplatin/paclitaxel as first therapy, 7 (23%) received ifosfamide/paclitaxel, 6 (19%) received a different regimen, and 3 (10%) did not receive chemotherapy. Patients treated with carboplatin/paclitaxel had a statistically significant longer PFS when compared to those receiving ifosfamide/paclitaxel (17.8 vs. 8.0 months, *p* = 0.025). OS was similar between all comparisons.

**Conclusions:**

In summary, in our cohort of ovarian carcinosarcoma patients, median PFS is longer in patients treated with carboplatin/paclitaxel compared to ifosfamide/paclitaxel. Overall survival was similar for all treatment groups, potentially due to subsequent treatment crossover. Given the rarity and aggressive nature of this tumor, further study into optimal first-line chemotherapy is warranted.

## Background

Ovarian carcinosarcomas, also known as malignant mixed Mullerian tumors (MMMT), represent 1% of all histologic subtypes of ovarian cancers and remain poorly understood [1]. While ovarian carcinosarcomas were once thought to be “collision tumors” with the presence of synchronous epithelial carcinoma and stromal sarcoma components, current data suggest they are de-differentiated carcinomas with cells that have a sarcomatous appearance and also cells with a carcinomatous histology [2]. Importantly, carcinosarcomas have been shown in multiple studies to be aggressive tumors, with one-third of patients having advanced disease at the time of diagnosis and a 60% overall recurrence rate [1, 3]. A review of the Surveillance, Epidemiology, and End Results (SEER) Program data from 1998 to 2009 demonstrated that patients with ovarian carcinosarcoma have consistently poorer prognosis than those with high-grade papillary serous carcinoma of the ovary. This study consisted of more than 14,000 ovarian cancer patients, approximately 9% of whom had ovarian carcinosarcoma, and the five-year disease specific survival rate was 28.2% for carcinosarcoma patients compared to 38.4% for high-grade papillary serous carcinoma patients (*p* < 0.001) [4]. Several additional single institution studies have also demonstrated that ovarian carcinosarcoma patients have lower response rates to chemotherapy and worse overall and disease-specific survival [4–7]. Given the low incidence of these tumors, prospective trials of chemotherapeutic approaches have been difficult to perform [8]. Thus, the preferred first-line chemotherapy for ovarian carcinosarcoma patients remains unknown.

Data guiding chemotherapy for the treatment of ovarian carcinosarcoma is largely extrapolated from studies on the treatment of uterine carcinosarcoma. Historically, ovarian carcinosarcomas were often treated with the same first-line chemotherapy as uterine carcinosarcomas, ifosfamide/paclitaxel [9, 10]. Additionally, multiple phase II studies investigating the use of carboplatin/paclitaxel in uterine carcinosarcoma demonstrated efficacy with this regimen [11, 12]. These studies have formed the basis of the Gynecologic Oncology Group (GOG) study 261, a phase III randomized, controlled trial comparing ifosfamide/paclitaxel to carboplatin/paclitaxel in the treatment of gynecologic carcinosarcomas, which allowed enrollment of patients with uterine or ovarian primary tumor sites.

Despite the above studies investigating chemotherapy treatment options for uterine carcinosarcoma, there are no published prospective studies looking at treatment options for ovarian carcinosarcoma. Small case series have reported on the efficacy of these and other regimens in the treatment of ovarian carcinosarcoma [5, 13–17]. We sought to determine the progression-free survival (PFS) and overall survival (OS) for patients with ovarian and primary peritoneal carcinosarcoma treated at our institution comparing different first-line chemotherapy regimens. Given the historical use of ifosfamide/paclitaxel and carboplatin/paclitaxel, we sought to compare these two regimens specifically as well as to compare platinum-containing regimens to non-platinum-containing regimens.

## Methods

This single-institution, retrospective study investigated patients with a pathologic diagnosis of ovarian, tubal, or primary peritoneal carcinosarcoma from September 1996 to July 2017. This study was approved by the University of Michigan Institutional Review Board (IRB # HUM00099459). Thirty-one patients with a histologic diagnosis of ovarian or primary peritoneal carcinosarcoma were identified (all cases were reviewed by a gynecologic pathologist). Baseline demographic information including age at diagnosis, stage, extent of cytoreduction (with optimal debulking defined as less than two centimeters of residual disease), and race were abstracted from each patient’s medical chart. First-line chemotherapy regimen was determined. Date of diagnosis was defined as date of primary debulking surgery or the date of diagnostic biopsy in the case of patients for whom debulking surgery was not undertaken. Progression-free survival (PFS) was defined as time from diagnosis to first evidence any of the following: appearance of new disease via radiographic imaging or clinical exam, elevation in CA125 above the normal range, or patient death from any cause. Overall survival (OS) was defined as months from diagnosis to patient death or last contact.

For statistical analysis, results were analyzed with standard descriptive and inferential statistical methods using SPSS Statistics 22. Mean age at diagnosis was compared using a one-way ANOVA. For categorical demographic variables, a chi-squared test was performed. Grubbs’ test for outliers was applied. Kaplan Meier curves were plotted for PFS and OS by initial chemotherapy regimen, and a log-rank Mantel-Cox test was used to compare PFS and OS between groups. Regression modeling was not performed due to small sample size. A *p*-value of < 0.05 was deemed statistically significant for all comparisons.

## Results

Thirty-one patients met inclusion criteria (Table 1). All patients were Caucasian race, and the mean age at diagnosis was 65.3 years (range 36–89 years). Twenty-nine patients had primary surgical debulking attempted, with optimal debulking achieved in 19 cases, suboptimal debulking in 9 cases and one case for which residual disease could not be determined from the medical record. Two patients did not undergo primary surgical debulking owing to their advanced stage and poor functional status at the time of diagnosis. With regard to stage at diagnosis, 7 patients were stage IC or II at diagnosis (22%), 21 patients were stage III (68%), and 3 patients were stage IV (10%). In our cohort, twenty-eight patients (90%) received chemotherapy. Fifteen patients (48%) received carboplatin/paclitaxel as their first chemotherapy, 7 patients (23%) received ifosfamide/paclitaxel, 6 patients (19%) received a different regimen and 3 patients (10%) did not receive chemotherapy. Only one patient (3%) received post-operative radiation.

**Table 1:** Demographic information by initial chemotherapy regimen with regard to age, stage, debulking surgery, and radiation

Demographic	All	No chemo	Carboplatin/ Paclitaxel	Ifosfamide/ Paclitaxel	Other Platinum	*p*-value
Total N	31	3 (10%)	15 (48%)	7 (23%)	6 (19%)	
Mean age at diagnosis (yrs ± St dev.)	65.39 ± 11.3	70.67 ± 7.6	68.33 ± 11.3	59.29 ± 4.4	62.50 ± 16.1	0.257
STAGE						
Stage IC-II	7 (22.6%)	0 (0%)	4 (26.7%)	2 (28.6%)	1 (16.7%)	0.736
Stage III	21 (67.7%)	3 (100%)	9 (60%)	4 (57.1%)	5 (83.3%)	
Stage IV	3 (9.7%)	0 (0%)	2 (13.3%)	1 (12.3%)	0 (0%)	
DEBULKING STATUS						
Debulking Surgery	29 (93.5%)	3 (100%)	14 (93.3%)	6 (85.7%)	6 (100%)	0.562
Optimal debulking	19 (61.3%)	1 (33.3%)	10 66.7%)	4 (57.1%)	4 (66.6%)	
Suboptimal debulking	9 (29.0%)	2 (66.7%)	4 (26.6%)	2 (28.6%)	1 (16.7%)	
Unknown Status	1 (3.2%)	0 (0%)	0 (0%)	0 (0%)	1 (16.7%)	
No Surgery Attempted	2 (6.5%)	0 (0%)	1 (6.7%)	1 (14.3%)	0 (0%)	
Radiation	1	0	0	0	1	NA

Table 1 legend: Categorical variables are presented as “n” (%). Continuous variables are presented as mean ± standard deviation. Patients in the “Other Platinum” category include those receiving Ifosfamide/Cisplatin, Carboplatin/Cytoxan, and Adriamycin/Cisplatin. *P*-values represent the following comparisons between the five chemotherapy groups: one-way ANOVA for mean age at diagnosis and chi-squared for stage, debulking status, and radiation. There were no significant differences between the chemotherapy groups for these variables.

Seventeen patients (55%) achieved a complete response, defined as no measurable evidence of disease on imaging and clinical exam following completion of primary chemotherapy. Eighteen patients (58%) were alive one year after diagnosis, 6 patients (19%) two years after diagnosis and 3 patients (10%) five years after diagnosis. The median PFS and OS by first-line chemotherapy regimen are presented in Table 2. For the entire cohort, the median progression-free survival was 9.3 months (95% CI: 3.7–14.9 months), and the median overall survival was 19.7 months (95% CI: 15.7–23.6 months). The one patient treated with cisplatin/Adriamycin (stage IIIC at diagnosis) had a PFS and OS of 213.2 months, and was excluded from further analysis after the application of Grubbs’ test for outliers (*p* < 0.05).

**Table 2:** Median progression-free survival and overall survival by initial chemotherapy regimen

First line chemotherapy	N	Median PFS (95% CI)	Median OS (95% CI)
No chemotherapy	3	1.3 (0.5–2.1)	1.3 (0.5–2.1)
Ifosfamide/Paclitaxel	7	8.0 (2.4–13.7)	19.0 (16.4–21.6)
Carboplatin/Paclitaxel	15	17.8 (4.0–31.6)	23.2 (22.0–24.4)
Ifosfamide/Cisplatin	4	13.0 (0.0–28.3)	20.6 (0.0–65.2)
Carboplatin/Cytoxan	1	13.4 (NA)	13.4 (NA)
Adriamycin/Cisplatin	1	213.2 (NA)	213.2 (NA)
Total	31	9.3 (3.7–14.9)	19.7 (15.7–23.6)

Table 2 legend: PFS and OS are reported for each chemotherapy regimen as “Median (95% confidence interval).”

**Fig. 1:**
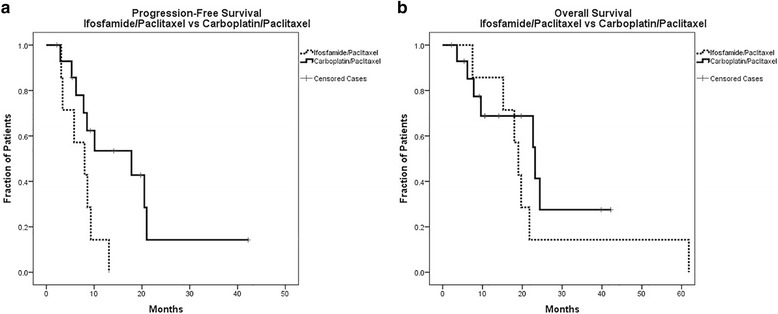
Progression-Free Survival and Overall Survival for Ifosfamide/Paclitaxel vs Carboplatin/Paclitaxel. **a** Kaplan Meier curve comparing PFS by initial chemotherapy: Ifosfamide/Paclitaxel compared to Carboplatin/Paclitaxel. Analyzed by log-rank Mantel-Cox test. *p* = 0.025. **b** Kaplan Meier curve comparing OS by initial chemotherapy: Ifosfamide/Paclitaxel compared to Carboplatin/Paclitaxel. Analyzed by log-rank Mantel-Cox test. *p* = 0.350

Next PFS and OS were directly compared by first-line chemotherapy regimen. Median progression-free survival for patients receiving no chemotherapy was 1.3 months (95% CI: 0.5–2.1 months). The three patients who did not receive chemotherapy (all stage IIIC at diagnosis) were all determined to have too poor of a performance status to receive chemotherapy. Given the common clinical use of chemotherapy doublets ifosfamide/paclitaxel and carboplatin/paclitaxel based on uterine carcinosarcoma literature, we sought to compare these two specific regimens in our ovarian carcinosarcoma cohort. Kaplan-Meier curves were plotted for PFS and OS for the two treatment groups and comparison carried out with a log-rank Mantel-Cox test (Fig. 1a and b). In comparing the outcomes for carboplatin/paclitaxel as a first-line regimen versus to ifosfamide/paclitaxel, those receiving carboplatin/paclitaxel had a median PFS of 17.8 months (95% CI: 4.0–31.6 months) compared to a median PFS of 8.0 months (95% CI: 2.4–13.7 months) for ifosfamide/paclitaxel (*p* = 0.025), representing a statistically significant improvement in PFS with carboplatin/paclitaxel as first-line chemotherapy (Fig. 1a). OS was similar between groups in this comparison with a median OS of 23.2 months (95% CI: 22.0–24.4 months) for carboplatin/paclitaxel and 19.0 months (95% CI: 16.4–21.6 months) for ifosfamide/paclitaxel (*p* = 0.350) (Fig. 1b).

**Fig. 2:**
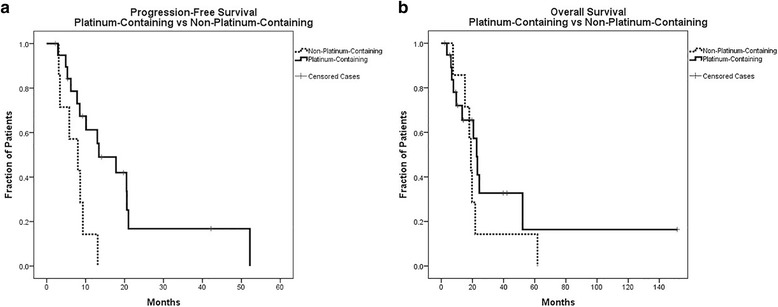
Progression-Free Survival and Overall Survival for Platinum-Containing vs Non-Platinum-Containing Regimens. **a** Kaplan Meier curve comparing PFS by initial chemotherapy: platinum-containing compared to non-platinum-containing. Analyzed by log-rank Mantel-Cox test. *p* = 0.008. **b** Kaplan Meier curve comparing OS by initial chemotherapy: platinum-containing compared to non-platinum-containing. Analyzed by log-rank Mantel-Cox test. *p* = 0.323

Kaplan-Meier curves were similarly plotted for PFS and OS for both platinum and non-platinum containing regimens (Fig. 2a and b), and they were compared with a log-rank Mantel-Cox test. In comparing platinum to non-platinum containing regimens, the median PFS for platinum-containing regimens was longer at 13.4 months compared to 8.0 months for non-platinum regimens (*p* = 0.008). Overall survival was similar between the two groups at a median of 22.7 months for platinum-containing regimens and 19.0 months for non-platinum-containing regimens (*p* = 0.323). Given the potential that the platinum component of the chemotherapy doublet is critical to the effectiveness of the regimen, we made one additional comparison: ifosfamide/cisplatin (*n* = 4) compared to carboplatin/paclitaxel (*n* = 15). There was no difference in median PFS (13.0 vs 17.8 months, *p* = 0.750) or OS (20.6 vs 23.2 months, *p* = 0.657) between these platinum-containing groups.

## Discussion

This retrospective study suggests that utilizing carboplatin/paclitaxel as first-line chemotherapy improves progression-free survival in patients with ovarian carcinosarcoma when compared to ifosfamide/paclitaxel. Interestingly, comparison of the patient population in our cohort treated with carboplatin/paclitaxel compared to ifosfamide/paclitaxel revealed that the carboplatin/paclitaxel patients were older (mean age of 68.3 years versus 59.3 years, *p* = 0.014) yet still had a longer PFS. Otherwise, there was no statistically significant difference between these two groups with regard to multiple variables: the percentage of patients that underwent debulking surgery (carboplatin/paclitaxel 93% versus ifosfamide/paclitaxel 86%, *p* = 0.34), the percentage in whom optimal debulking was achieved (71% versus 65%, *p* = 0.83), distribution of stage (60% stage III vs 57%, *p* = 0.736), or the percentage in whom complete response was achieved following initial therapy (80% vs 57%, *p* = 0.26) (Table 1).

No treatment approach queried demonstrated a superior overall survival as compared to other treatment options. We hypothesize that this is due to subsequent treatment cross-over following progression or recurrence, such that many patients receive all of the potentially active chemotherapy agents at some time during their treatment. Due to post-progression therapy impacting OS in the treatment of ovarian cancer, it has been proposed that PFS be used as a valid endpoint for the assessment of treatment efficacy [18].

The relative rarity of ovarian carcinosarcoma has limited the ability to perform prospective trials assessing chemotherapy options. A Cochrane review of chemotherapy and/or radiotherapy in combination with surgery for ovarian carcinosarcoma found no evidence to inform decisions about neoadjuvant or post-operative chemotherapy for women with ovarian carcinosarcoma [19]. Additionally, data from GOG-261, which allowed for enrollment of patients with ovarian carcinosarcoma in addition to uterine carcinosarcoma, is not yet available, and we do not know yet how many ovarian carcinosarcoma patients were enrolled in the study. Thus, we are currently limited to retrospective studies to guide treatment approaches. Multiple small studies have investigated prognosis and treatment options for ovarian carcinosarcoma (Table 3).

**Table 3:** Key retrospective studies of chemotherapy regimens in patients with ovarian carcinosarcomas

Study	Citation	N	Median PFS (mo)	Median OS (mo)	Findings	Regimens Compared
Brown, E. et al.	[6]	65	6.4	8.2	Ovarian carcinosarcomas are associated with poorer response to chemotherapy, PFS and disease specific survival compared to high grade papillary serous carcinoma of the ovary. Debulking status significantly impacts outcomes.	No
Kanis, M.J. et al.	[13]	28	10	21	No difference in PFS and OS between patients treated with carboplatin/paclitaxel and those treated with other first-line chemotherapy regimens. Optimal debulking improves PFS.	Yes
Rutledge, T.L. et al.	[14]	31	ND	21	Advanced stage disease worsens PFS. PFS is better in ifosfamide/cisplatin compared to carboplatin/paclitaxel. OS similar between the two chemotherapy regimens.	Yes
Silasi, D.A. et al.	[15]	22	6–13	38	PFS is the same for ifosfamide/cisplatin compared to carboplatin/paclitaxel.	Yes
Rauh-Hain, J.A. et al.	[17]	50	11	24	Ovarian carcinosarcomas are associated with poorer response to platinum-based chemotherapy, PFS and disease specific survival compared to high grade papillary serous carcinoma of the ovary.	No
Cicin, I. et al.	[19]	26	ND	26	Median survival is longer with early versus late stage disease. Adjuvant platinum-based chemotherapy is predictive of better outcome.	Yes
Leiser, A.L. et al.	[20]	30	12	43	Median PFS is 12 months when treated with combination of a platinum and taxane.	No
Brackmann, M. et al.	–	31	9.3	19.7	Longer PFS with carboplatin/paclitaxel compared to ifosfamide/paclitaxel. OS similar between comparison groups.	Yes

Table 3 legend: Studies are listed in the order they appear in the text. For each study, the number of participants, median PFS and OS, as well as key findings are listed. If the study compared different chemotherapy regimens directly, it is listed. “ND” = not determined.

Although several of these studies attempted to compare different potential chemotherapy regimens, no definitive preferred first-line treatment has emerged. In the few studies where direct comparisons between regimens were made, results were mixed. In one study, PFS was longer in patients treated with ifosfamide/cisplatin compared to carboplatin/paclitaxel [14]. However, in another, PFS was the same when comparing ifosfamide/cisplatin to carboplatin/paclitaxel [15]. Additionally, no study prior to our work directly compares ifosfamide/paclitaxel to carboplatin/paclitaxel, an especially relevant comparison given the frequent extrapolation of uterine carcinosarcoma treatment regimens to the care of women with ovarian carcinosarcoma [9, 10]. Our study adds to the published literature by addressing this comparison. Moreover, our data further lend support to the first-line use of platinum-based regimens in ovarian carcinosarcoma, consistent with our evolving understanding of carcinosarcomas as epithelial malignancies and previous reports supporting this approach [16, 20–25]. Given the lower morbidity of carboplatin/paclitaxel in comparison to regimens containing ifosfamide, cisplatin, or both, our comparison is of particular interest. Our work is similar in sample size to previously reported studies and PFS and OS are in line with published data.

Our study has similar limitations to previously published work, including overall low patient numbers, the retrospective nature of the work, the non-randomized treatment groups (including some shift in chemotherapy preference over time) and the high crossover rate with subsequent therapies that confounds interpretation of overall survival. Additionally, several patients in the cohort represent recent diagnoses and their survival data is not yet mature. We plan an additional analysis of these patients in the future.

As our understanding of tumor biology expands, continued molecular characterization of ovarian carcinosarcoma is warranted to define both targetable alterations and predictive biomarkers of response. Significant progress was made in this regard with the finding that ovarian and uterine carcinosarcomas represent metaplastic carcinomas, therefore suggesting traditional regimens for carcinomas would be favored over sarcoma regimens [2]. These data support the biologic rationale for treating ovarian carcinosarcoma patients with carboplatin/paclitaxel, consistent with preferred treatment for ovarian carcinomas. Detailed molecular analysis of ovarian carcinosarcomas remains lacking at this time. Studies are generally still performed with both ovarian and uterine carcinosarcomas to increase sample size, although it is unclear whether there is a molecular rationale for this approach. It is known, however, that altered expression of p53 is frequent in gynecologic carcinosarcomas [26]. A recent molecular characterization of an ovarian carcinosarcoma patient-derived xenograft revealed p53 and phosphoinositide-3-kinase, catalytic, alpha polypeptide (PIK3CA) mutations, as well as epidermal growth factor receptor overexpression, vascular endothelial growth factor receptor C overexpression and activation of the insulin-like growth factor pathway [27]. Further studies are warranted to determine the frequency of both germline and somatic genetic alterations in ovarian carcinosarcoma patients and the potential for such mutations to inform clinical treatment decisions.

## Conclusions

In conclusion, ovarian carcinosarcomas are rare, aggressive tumors with poor prognosis. The optimal first-line chemotherapy for these patients remains unknown. In our study, patients treated with carboplatin/paclitaxel have a longer PFS than patients treated with ifosfamide/paclitaxel. Moreover, platinum-containing regimens appear to prolong PFS when compared to non-platinum containing regimens. Overall survival was similar for all treatment groups, likely due to subsequent treatment crossover at the time of recurrence. Given lower morbidity, lower cost and fewer hospital days associated with carboplatin/paclitaxel regimens as well as longer PFS, we favor this first-line chemotherapy for ovarian carcinosarcomas after primary surgical debulking. Further study into optimal chemotherapy remains warranted, including cooperative clinical trials and continued molecular characterization of ovarian carcinosarcomas.

## Abbreviations

GOG: Gynecologic Oncology Group
MMMT: Malignant mixed Mullerian tumors
ND: Not determined
OS: Overall survival
PFS: Progression-free survival
PIK3CA: Phosphoinositide-3-kinase, catalytic, alpha polypeptide
SEER: Surveillance, Epidemiology, and End Results
